# Beneficial Effects of Brief Alcohol Interventions on Diet, Physical Activity and Body-Mass Index: Secondary Outcome Data from a Randomized Controlled Trial Among General Hospital Patients

**DOI:** 10.3390/healthcare14213590

**Published:** 2026-10-23

**Authors:** Anika Tiede, Sophie Baumann, Filipa Krolo-Wicovsky, Gallus Bischof, Beate Gaertner, Ulrich John, Jennis Freyer-Adam

**Affiliations:** 1 Department of Prevention Research and Social Medicine, Institute for Community Medicine, University Medicine Greifswald, Walther-Rathenau-Str. 48, 17475 Greifswald, Germany; anika.tiede@med.uni-greifswald.de (A.T.); filipa.krolo-wicovsky@med.uni-greifswald.de (F.K.-W.); ulrich.john@med.uni-greifswald.de (U.J.); 2 Institute for Medical Psychology, University Medicine Greifswald, 17475 Greifswald, Germany; 3 German Centre for Cardiovascular Research, 17475 Greifswald, Germany; 4 Department of Methods in Community Medicine, Institute for Community Medicine, University Medicine Greifswald, 17475 Greifswald, Germany; sophie.baumann@med.uni-greifswald.de; 5 Department of Psychiatry and Psychotherapy, University of Lübeck, 23538 Lübeck, Germany; gallus.bischof@uksh.de; 6 Department of Epidemiology and Health Monitoring, Robert Koch Institute Berlin, 13353 Berlin, Germany; gaertnerb@rki.de

**Keywords:** health risk behaviors, overweight, body-mass index, diet, alcohol, smoking, physical activity, screening and brief intervention, motivational interviewing, digital

## Abstract

**Highlights:**

**What are the main findings?**

- Brief behavior change interventions targeting alcohol use among proactively recruited general hospital patients resulted in a lower increase in body-mass index after 24 months.
- Findings from the three-arm randomized controlled trial were similar for in-person counseling and computer-generated feedback.

**What is the implication of the main finding?**

- Proactive, theory-driven interventions that target alcohol use only can also positively impact body-mass index, an indicator of frequently co-occurring overweight.
- To achieve more comprehensive changes for disease prevention, individually tailored interventions directly targeting all co-occurring behavioral risk factors still seem to be required.

**Abstract:**

Background: Co-occurrence of behavioral risk factors (BRFs) such as tobacco smoking, at-risk alcohol use, unhealthy diet, insufficient physical activity and/or overweight is crucial concerning overall morbidity and mortality risk and is highly prevalent among healthcare patients, e.g., 93% of general hospital patients with at-risk alcohol use also report at least one further BRF. This study aimed to investigate whether two types of brief intervention targeting alcohol use, i.e., behavior change counseling and individualized computer-generated feedback, that have been found to be effective concerning alcohol use reduction and general health measures, produced additional beneficial effects concerning healthy diet, physical activity, and body-mass index among general hospital patients over two years after hospitalization. Methods: The randomized controlled trial included 960 systematically identified hospitalized patients with at-risk alcohol use allocated to three groups: in-person counseling, computer-generated written feedback, and assessment only. Both alcohol interventions included contacts at baseline, month 1, and month 3. Latent growth models analyzed secondary intervention effects concerning self-reported vegetable/fruit intake, physical activity, and body-mass index over 6, 12, 18, and 24 months. Results: At month 24, body-mass index was less strongly increased after in-person counseling versus assessment only (*p* = 0.022, Cohen’s d = 0.77). Borderline significant effects indicated similar changes for computer-generated feedback (*p* = 0.050, d = 0.64) and for increased physical activity (*p*_in-person_ = 0.052, d = 0.31). Conclusions: Brief alcohol interventions proactively delivered to general hospital patients admitted for any disease or injury also resulted in significant and borderline significant medium-sized effects concerning body-mass index. To achieve more comprehensive changes for the prevention of non-communicable diseases, individually tailored interventions targeting all co-occurring BRFs still seem to be required.

## 1. Introduction

Concerning the prevention of noncommunicable diseases, the co-occurrence of behavioral health risk factors (BRFs), namely at-risk alcohol use, tobacco smoking, unhealthy diet, and insufficient physical activity, constitutes a major issue for at least two reasons. Firstly, BRFs account for a large impact on the global burden of noncommunicable diseases and are the leading causes of premature death globally, and their co-occurrence may not only have an additive but more than a multiplicative effect on disease incidence or mortality [1,2,3,4]. Secondly, BRF co-occurrence is highly prevalent, affecting more than half of the general population [5,6,7]. For example, among adults with at-risk alcohol use, about 90% report one or more further BRF(s), as shown for the general population and for general hospital patients [6,8]. That is, general hospital patients with at-risk alcohol use also report insufficient physical activity (63%), overweight indicated by body-mass index equal to or larger than 25.0 (58%), and/or tobacco smoking (49%) [8].

In terms of the prevention and treatment of chronic diseases, hospitalization may be considered a “learnable” moment and is often associated with higher behavior change motivation [9,10]. However, a recent study revealed that general hospital patients with at-risk alcohol use reduced substance use but reported less physical activity and an increasing body-mass index over two years after hospitalization [11]. These findings are consistent with those of a cross-sectional nationwide study which revealed that, for every additional year of age, the proportions of adults who smoke and/or drink alcohol at risk decrease, while the proportions of overweight and/or physically inactive adults increase [6]. Thus, hospitalization alone may not be related to sufficient and comprehensive behavior change in this regard, and additional behavior change interventions are needed to achieve positive and comprehensive behavioral changes.

Brief alcohol interventions (BAIs), i.e., brief advice or counseling of less than six sessions and less than 1 h can successfully reduce alcohol use in healthcare patients [12,13]. A three-arm randomized controlled trial (RCT) revealed that motivational interviewing-based behavior change counseling as well as theory-driven computer-generated feedback delivered to proactively recruited general hospital patients with at-risk alcohol use were found to improve health measures beyond alcohol use. To be more precise, the proportion of participants drinking at-risk was reduced by 50% at month 6 as a result of in-person counseling. Computer-generated feedback reduced the primary outcome of grams of alcohol consumed per week up to month 24. Intervention effects concerning self-rated health, mental well-being, and the likelihood of having sick days were found to be medium to large, increasing over two years [14,15,16]. These findings are encouraging as they seem to be achievable in hospital patients with little intention to change their alcohol use when proactively approached [17]. However, in terms of multiple co-occurring BRFs, it seems warranted to investigate whether BAIs may also positively affect co-occurring energy-balance-related BRFs such as unhealthy diet, insufficient physical activity, and overweight. Such additional beneficial side effects or spillover effects have rarely been investigated but are expectable for various reasons: (1) Counseling for one BRF could increase awareness to rethink other lifestyle choices as well. (2) Successfully changing one BRF may positively affect self-efficacy to change other BRFs as well [18,19]. (3) Reduced alcohol intake is expected to be accompanied by lower caloric intake, which could positively impact body-mass index, a widely used indicator of overweight, resulting from an imbalance between energy intake (diet) and energy outtake (physical activity). The few studies that investigated beneficial effects with regard to other BRFs revealed rather mixed and only a few promising findings. Neither an RCT conducted among patients with traumatic brain injury nor a meta-analysis among adults revealed beneficial BAI effects concerning nutrition and physical activity or tobacco smoking, respectively [20,21]. The above-mentioned three-arm RCT among general hospital patients revealed no beneficial BAI effect concerning reduced tobacco smoking for an in-person-delivered BAIs but for computer-generated feedback. Up to month 6, those at-risk alcohol users who also smoked tobacco reduced the number of cigarettes more strongly following feedback [22]. Thus, given that digital interventions are being developed and implemented in healthcare [23], it may be worthwhile to investigate whether potential beneficial BAI effects concerning energy-balance related BRFs occur for either way of BAI delivery, that is for in-person delivery and for individualized computer-generated feedback.

Using data from the above-described RCT, this study’s aim was to analyze whether proactive BAIs which have been shown to reach the majority of general hospital patients with at-risk alcohol use [17], to reduce alcohol use [15], to reduce tobacco smoking in the short term, and to improve indicators of health over two years after hospitalization [14,16], also improved vegetable and fruit intake, physical activity, and body-mass index. In addition, the study aimed to investigate whether such additional beneficial effects could be observed for in-person delivery and for computer-generated written feedback.

## 2. Methods

Data from the three-arm RCT “Testing delivery channels of individualized motivationally tailored alcohol interventions among general hospital patients: in-person vs. computer-based, PECO” were analyzed (ClinicalTrials.gov: NCT01291693 [15,17,24]); pre-registered in February 2011. The ethics committee of the University Medicine Greifswald approved the study prior to data collection (BB07/10, BB105/13). All trial participants provided written informed consent. Vegetable and fruit intake, physical activity, and overweight status were not pre-specified outcomes of the investigated BAIs. However, given various positive intervention effects [14,15,16], the exploration of potential beneficial effects concerning energy-balance-related BRFs appears to be warranted. Patients and public were not involved in the design, conduct, and reporting of the trial.

### 2.1. Sampling Frame and Participants

Participants were recruited on 13 wards of five medical departments (internal medicine [cardiology, angiology], internal medicine [gastroenterology, nephrology, endocrinology], otorhinolaryngology, trauma surgery, general, and thorax surgery) at the University Medicine Hospital Greifswald, Germany. Between February 2011 and July 2012, trained research assistants approached all 18–64-year-old consecutively admitted patients on each working day. Patients admitted over weekends or holidays were approached on the next working day. In cases where patients were unavailable or feeling unwell, several contact attempts were performed. Patients were asked to respond to an electronic self-administered questionnaire on health behaviors. Patients who were cognitively or physically incapable or terminally ill, with highly infectious diseases, discharged or transferred outside the study area within the first 24 h, already recruited for the study during an earlier hospital stay, with insufficient German language skills, or employed at the conducting research institute were excluded. Those patients who screened positive for at-risk alcohol use according to national guidelines [25] were eligible for trial inclusion. At-risk alcohol use was assessed by the Alcohol Use Disorder Identification Test (AUDIT)–Consumption with cut-off values of ≥ 4/≥5 for women/men [26]. Patients with total AUDIT scores ≥ 20 [27] were excluded due to lack of efficacy of BAIs in persons with more severe alcohol problems [28]. Patients with no telephone were also excluded.

**Figure 1:**
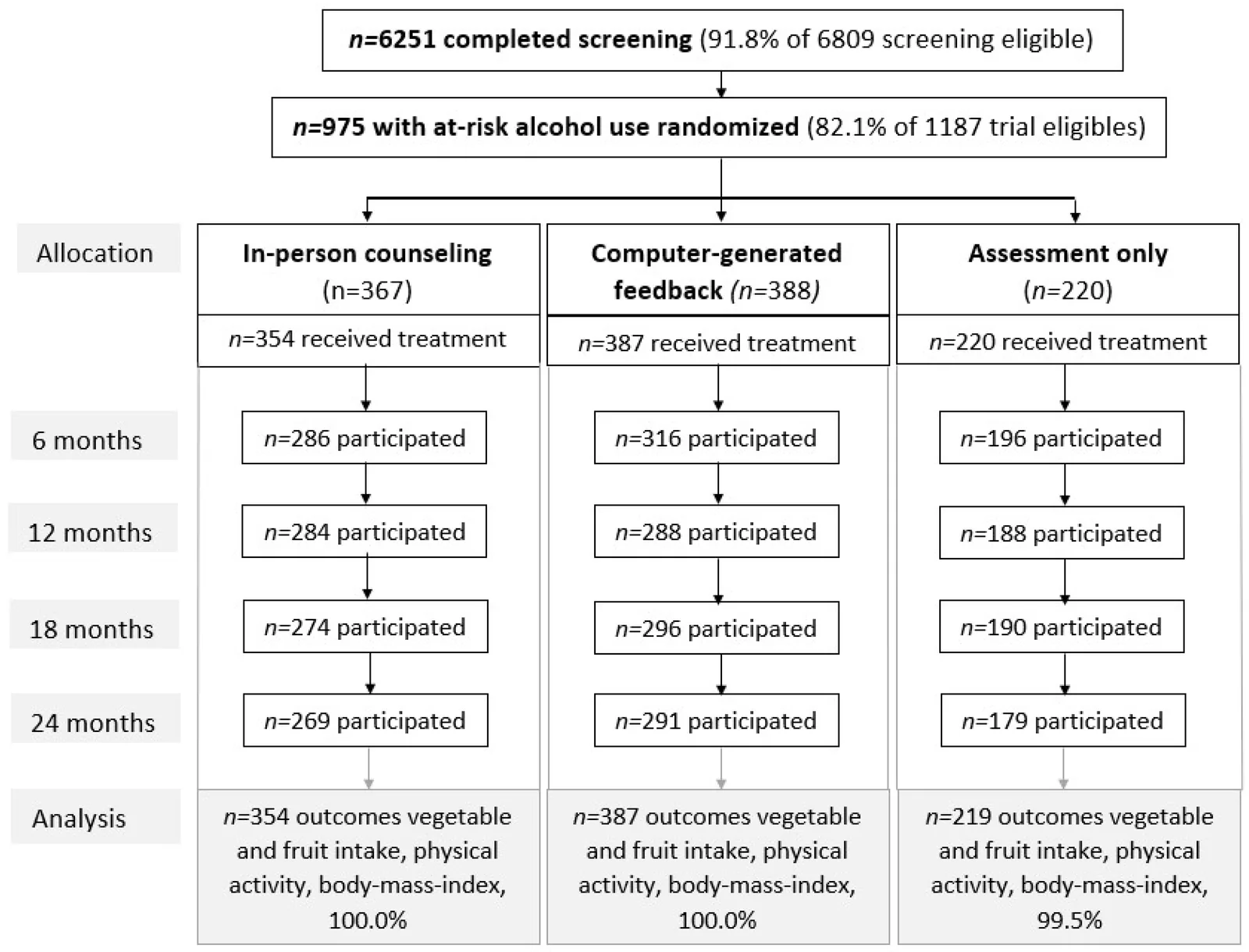
Participant flow stratified by study group.

Among the 6809 patients meeting the screening inclusion criteria, 6251 (91.8%) completed the screening. Of these, 1188 patients with at-risk alcohol use were eligible for trial inclusion. Of these, 975 agreed to participate, and 961 (81%) received their allocated intervention: in-person counseling, computer-generated feedback, or assessment only (Figure 1). To prevent study groups from exchanging intervention information, study group allocation rotated weekly across wards. Different from cluster-randomized trials, no severe loss of power was expected as all wards provided participants for each study group, a large number of 140 clusters and a small average number of participants per cluster (*n* = 7), were obtained [29]. An allocation ratio of 2:2:1 (in-person counseling: computer-generated feedback: assessment only) was required to detect small intervention effects of Cohen’s d = 0.33 and small between-intervention effects of d = 0.23 concerning the primary target behavior alcohol use reduction. Recruitment was stopped as soon as the expected sample size of 975 participants was achieved. More details may be found in the Supplementary Material and elsewhere [15].

### 2.2. Intervention

As reported in more detail elsewhere [15,17,24], both BAIs tested were designed to be comparable concerning content. They were individually tailored, driven by behavior change theory, and included three intervention contacts at months 0, 1, and 3, as well as normative and ipsative feedback. The normative feedback was based on the participant’s data compared to those of others. The ipsative feedback was based on the participant’s changes over time. Both interventions were proactive, i.e., all patients were contacted personally on the ward and by phone at months 1 and 3. Prior to feedback delivery, self-report data on current alcohol use and the four dimensions of the transtheoretical model of intentional behavior change [30] were assessed, i.e., stage of change, processes of change, decisional balance, and self-efficacy. This was either self-administered at month 0 or conducted via standardized computer-assisted telephone interviews at months 1 and 3. According to the model, persons progress through stages of change, i.e., from not thinking about change (precontemplation), through being ambivalent about change (contemplation), planning change (preparation), manifesting change (action), to maintaining change (maintenance). Interventions are expected to be most effective when tailored to a person’s current stage [30]. In this trial, both interventions were matched to the current stage using all four dimensions of the underlying theory, a prerequisite to achieve larger intervention effects [31,32]. Both interventions primarily differed in the way of delivery.

In-person counseling on alcohol use was primarily delivered by psychologists trained in motivational interviewing-based behavior change counseling [33], face-to-face at baseline, and by phone at months 1 and 3. Overall, summed-up across all three consultations, participants received 35 min of counseling (median). Eighty-three percent of the participants were counseled 2–3 times. Counseling was delivered with acceptable adherence to motivational interviewing [33]. For more details, see elsewhere [15,17].

Computer-generated written feedback on alcohol use was delivered through individually tailored 3- to 4-page letters generated by a computer expert system programmed in Microsoft Office Access 2007 (Microsoft Corporation, Redmond, U.S.A.). The letters provided stage-tailored feedback as described above, were written in a patient-accepting, supportive and non-confrontational style, and were supported by stage-matched manuals. These were delivered either on the ward at baseline or by regular mail at months 1 and 3. Eighty-nine percent of the participants received 2–3 feedbacks [17].

Assessment only consisted of routine hospital care and minimal assessment at baseline only.

### 2.3. Follow-Ups

After 6, 12, 18, and 24 months, computer-assisted telephone interviews assessed self-report outcomes. Follow-ups were conducted in 2011–2014. Incentives in the form of vouchers were paid before, at month 12 (5 €), or after participation, at months 6, 18, and 24 (10/15/20 €). Overall follow-up participation per time-point was 77–83% among all 961 baseline participants (Figure 1). Outcome assessors were not informed about group allocation as described in more detail elsewhere [15].

### 2.4. Measurements

#### 2.4.1. Outcomes

Vegetable and fruit intake, a simple, widely used and suitable indicator of diet [34] was determined at months 0, 6, 18, and 24, using the question “How many servings of fruit and vegetables do you usually eat per day?” with six response categories: 0, 1, 2, 3, 4 and 5 or more servings of vegetables and fruit per day. Five or more servings were counted as 5 (*n* = 40).

Physical activity was assessed at months 0, 6, 18, and 24, using the question “Do you also do sports?” with six response categories (0–5): none, <1, 1–2, 2–3, 3–4 and >4 h per week as applied elsewhere [11].

Body-mass index was obtained from self-reported weight in kilogram at months 0, 6, 12, 18, and 24, baseline height in meters and the formula bodyweight (kg)/body height (m)^2^.

Harms were not systematically assessed.

#### 2.4.2. Descriptives

Socio-demographics included sex (female/male), age (in years), living in a partnership (yes, including being married/no), level of school education (low [<10 years]/medium [10–11 years]/high [>11 years of school]), employment status (employed /unemployed/other), and medical department to which participants were admitted (internal medicine/general surgery/trauma surgery/ear–nose–throat).

Substance use-related characteristics included alcohol use and problem severity assessed by the AUDIT total score (range: 0–19) [35]. The motivational stage to reduce or to abstain from alcohol drinking was assessed by a 4-item staging algorithm (precontemplation/contemplation/preparation/action), an adaption [17] from previous measures [36]. Co-substance use was obtained by the number of cigarettes currently smoked per day. Never-smokers and former smokers were both deemed to be equivalent to smoking 0 cigarettes per day.

Health-related characteristics included self-rated general health and mental health. General health was assessed using the single-item question “Would you say your health in general is: excellent (4)/very good (3)/good (2)/fair (1)/poor (0)?” [37]. This is an independent and reliable predictor of mortality [38]. Mental health was assessed by the five-item mental health inventory with higher scores indicating better mental health [39].

### 2.5. Statistical Analyses

The statistical analysis plan was developed before data analysis following previous outcome analyses [14,15,16]. To investigate additional beneficial effects of both ways of delivering BAIs on vegetable and fruit intake, physical activity and body-mass index, latent growth models [40] were applied using Mplus 8.4 Muthén & Muthén, Los Angeles, U.S.A. [41]. In a latent growth model, repeated measures of the outcome variables are treated as observed indicators of latent growth factors representing outcome growth trajectories. In our study, rescaled likelihood ratio tests spoke in favor of linear models for all outcomes. Thus, the observed indicators number of servings of vegetables and fruit per day, amount of physical activity, and body-mass index were regressed on the growth factors using linear regression models. The ordinal response categories assessing the amount of time spent on physical activity were treated as a continuous measure as they represent increasing levels of the underlying continuous construct of the amount of time spent. Furthermore, variances of intercepts and slopes were freely estimated. To provide a detailed picture of trajectories of change over time, differences in means in change from baseline to follow-up and 95% confidence intervals (CIs) for each follow-up were calculated. To reduce the number of comparisons, statistical significance (*p* < 0.05) was tested for the primary time-point at month 24 only. Given the explorative nature of the analyses, no further statistical correction for multiple comparisons was used. For significant and borderline significant (*p* = 0.05) comparisons, Cohen’s d for standardized slope differences was provided as an indicator of effect size (0.2 to <0.5 small, 0.5 to <0.8 medium, ≥0.8 large).

A maximum likelihood estimator with robust standard errors was used. Models were estimated under a missing at random assumption using all available data, allowing us to include all participants in each analysis, regardless of follow-up participation. Models were adjusted for (a) all baseline characteristics related to follow-up participation in multivariate models (*p*s ≤ 0.1), i.e., age, school education, AUDIT, motivation to change, and mental health, to account for potential differential attrition effects and to make the missing at random assumption more plausible (for further details, see [15]); (b) medical department to prevent potential clustering effects; (c) gender and employment status to account for chance imbalances between study groups (*p*s ≤ 0.1); and (d) cigarettes per day and subjective general health to account for the potential relevance of co-substance use and current health status. Comparative fit indices ranging between 0.91 and 0.99 and root mean square error of approximation ranging between 0.01 and 0.05, indicated adequate fits of the three models to the data. One participant was excluded from the analyses due to missing baseline covariate motivational stage of change, leaving n = 960 participants for the analysis.

## 3. Results

### 3.1. Sample Characteristics

The mean age of the sample was 40.9 years at baseline (standard deviation, SD = 14.1). Of the sample, 74.9% (*n* = 719) were male, 24.7% (*n* = 237) reported 12 or more years of school education, 65.2% (*n* = 626) were employed, 68.2% (*n* = 654) living in a partnership; and 31.0% (*n* = 298) admitted to the internal medicine wards, 11.8% (*n* = 113) to general and thorax surgery, 34.0% (*n* = 326) to trauma surgery, and 23.2% (*n* = 223) to otorhinolaryngology wards. The mean AUDIT-Consumption score at baseline was 6.0 (SD = 1.6). The mean number of servings of vegetables and fruits was 2.0 (SD = 1.2), the mean body-mass index was 26.9 (SD = 5.4); and 49.3% (*n* = 473) reported no additional sports. These outcome variables stratified by study group and over time are reported in Table 1. Other baseline characteristics including all covariates stratified by study group may be found elsewhere [15]. Trial participants differed from non-participants with regard to younger age and higher school education [17].

### 3.2. Vegetable and Fruit Intake

**Figure 2:**
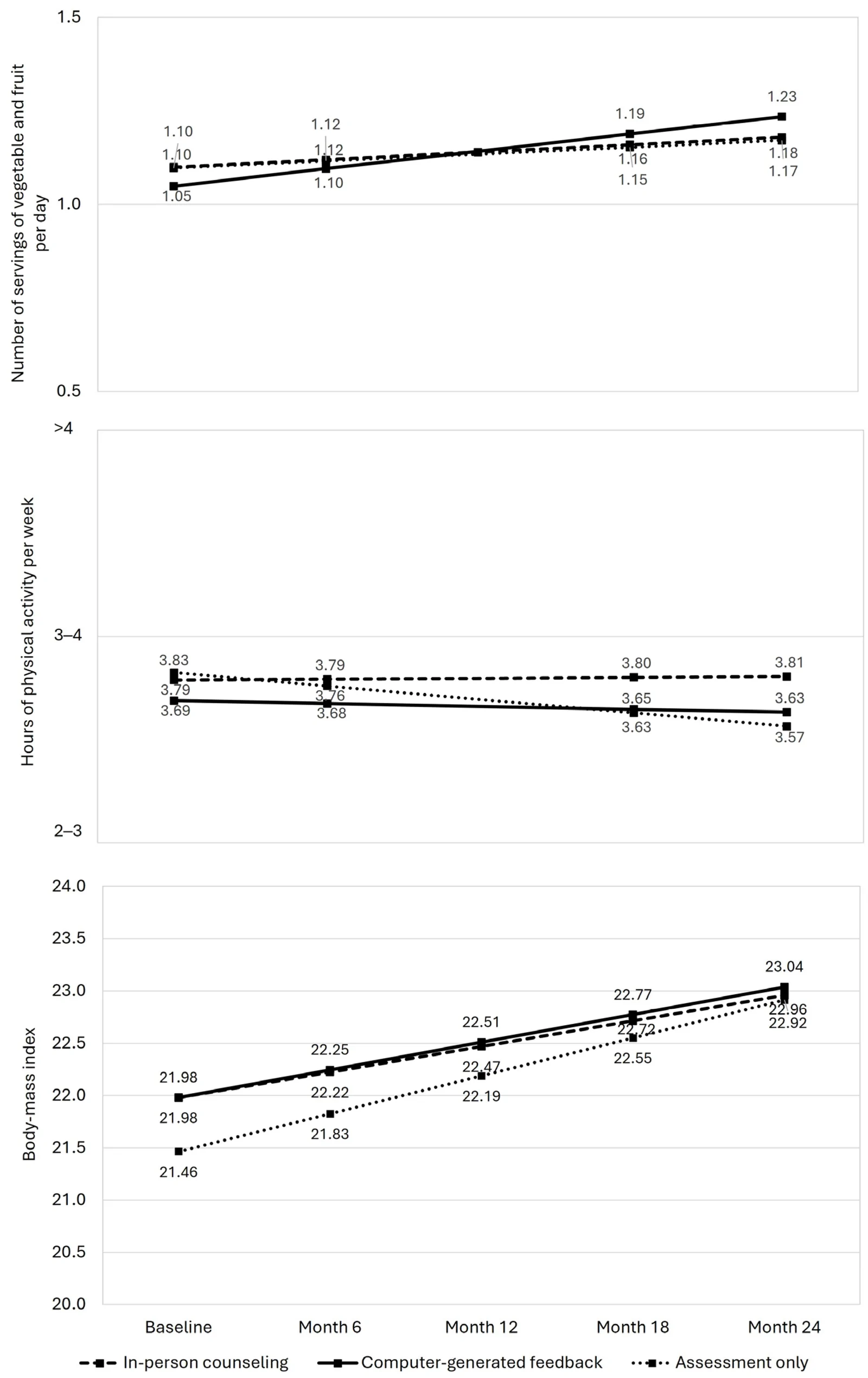
Model-implied values of vegetable and fruit intake, physical activity, and body-mass index over 24 months by study group.

Compared to baseline, none of the three study groups significantly changed vegetable and fruit intake between baseline and month 24 (*p*s ≥ 0.631, Figure 2). No statistically significant differences in change were found between any of the two intervention groups and assessment only and between intervention groups (*p*s ≥ 0.261, Table 2).

### 3.3. Physical Activity

In all three groups, the amount of physical activity per week at month 24 was not significantly different from baseline (*p*s ≥ 0.572, Figure 2). In comparison to assessment only, there was a borderline significant effect indicating a slightly greater increase in physical activity in the in-person group by 0.07 with every 6 months, resulting in a mean difference in change between groups of 0.278 at month 24 (95% CI: −0.002; 0.56, *p* = 0.052, d = 0.31). No significant differences in changes to physical activity were found between the computer-generated feedback group and the assessment-only group, or between the intervention groups (*p*s ≥ 0.129, Table 2).

### 3.4. Body-Mass Index

In the assessment only group, the body-mass index increased significantly by 0.36 every six months (95% CI 0.05; 0.68, *p* = 0.025, Figure 2). In both intervention groups, the body-mass index did not increase significantly by month 24 (*p*s ≥ 0.101). In comparison to assessment only, the body-mass index increased less strongly after in-person counseling (mean difference in change with every 6 months: −0.119), resulting in a mean difference in change between both groups of −0.475 at month 24 (95% CI −0.88; −0.07, *p* = 0.022, d = 0.77, Table 2). The results also indicated a borderline significant effect toward a smaller increase in body-mass index after computer-generated feedback (mean difference in change with every 6 months: −0.099), resulting in a mean difference in change in contrast to assessment only of −0.396 at month 24 (95% CI −0.79; 0.00, *p* = 0.050, d = 0.64). No significant difference in change between intervention groups was found (*p* = 0.650, Table 2).

## 4. Discussion

This study investigated potential beneficial effects of BAI on diet, physical activity, and overweight status among general hospital patients with at-risk alcohol use over two years. The three main findings are: Firstly, beneficial BAI effects concerning body-mass index were found for in-person counseling and for computer-generated written feedback, when taking borderline significant effects into account. Secondly, a borderline significant effect on physical activity was found for in-person counseling. Thirdly, no significant differences in change between both intervention groups were observed.

To our knowledge, little is known about beneficial side effects of BAI on energy-balance-related BRFs so far. The lower increase in the body-mass index after having received in-person counseling, and borderline significance after having received computer-generated written feedback is likely to be related to reduced caloric intake due to reduced alcohol use, as previously reported [15]. However, it may also be related to the small (albeit only borderline significant) effect of in-person counseling on increased physical activity. The intervention or reducing alcohol use may have enhanced general self-efficacy in participants to change physical activity as well [19]. As mediation was not tested, further research is required to shed more light on actual mechanisms. Taken together, these additional beneficial medium-sized effects concerning accompanying BRFs with Cohen’s d values between 0.31 and 0.77 support the medium to large beneficial health effects found for these BAIs previously [14,16]. Although some effects were small or only borderline significant, they seem quite noteworthy given the briefness of the interventions targeting an entirely different behavior, the patients’ low initial motivation to change the target behavior [17], and taking into account that even small intervention effects in large populations may have a substantial impact on public health [42]. In this regard, multiple small BRF changes may be clinically relevant in terms of the prevention and treatment of noncommunicable diseases.

Our null-findings concerning beneficial BAI effects with regard to vegetable and fruit intake are in line with previous findings revealing no beneficial BAI effects concerning tobacco smoking [21]. A couple of explanations may be considered. Firstly, the simple measure used may have been rather insensitive to behavior change as discussed below. Secondly, single behavior change interventions such as BAIs may be insufficient to achieve behavioral changes in all co-occurring BRFs and sustained change in lifestyle. For example, important predictors of change in vegetable and fruit intake such as knowledge about vegetable and fruit intake, self-efficacy, and social support [43] were not addressed as part of the BAI.

Also, in line with other primary and secondary outcome analyses of the same trial [14,15,16,22], beneficial BAI effects concerning the three co-occurring BRFs did not vary significantly by way of intervention delivery. This supports our previous conclusion that electronically delivered interventions may be an effective and low-cost complement to in-person delivered interventions, particularly for the large subgroup of patients with lower problem load [44]. Although the medium-sized effect of the computer-generated feedback concerning body-mass index was only borderline significant, it may be explained by certain advantages of computer-generated feedback or electronic delivery: (a) it allows reliable processing and storing of data, (b) participants may feel less stigmatized and more autonomous as the addressed behavior is not raised by a person and as they may decide themselves if and when intervention content is looked up.

Strengths of this study include firstly the first investigation of potential side or spillover long-term effects of two ways of delivery of BAIs concerning energy-balance-related BRFs. Secondly, selection bias was rather low in this study. Due to proactive recruitment, the vast majority of eligible patients (81%) participated in the intervention study and provided follow-up data at each time-point (80% ± 3%). In addition, maximum likelihood estimation allowed inclusion of all participants in the analyses regardless of missingness at follow-up. Thirdly, given low selection bias, even patients with initially low motivation to change and low alcohol use severity were successfully reached [17]. This is particularly noteworthy given that prevention efforts often fail to reach people who are not yet ready to change their behavior; and given that our study highlights that behavioral changes are possible among those who were initially not yet ready, even beyond the target behavior itself. The application of the transtheoretical model of intentional behavior change allowed to provide stage-tailored assistance which patients in different motivational stages might psychologically require [30]. Fourthly, self-report bias resulting from socially desirable answers may not severely diminish the results of this study. Intervention participants may be less prone to provide socially desirable answers [45] regarding secondary outcomes such as body-mass index. Other than the primary outcome alcohol use, these outcomes remained completely unaddressed as part of the intervention on alcohol use. Thus, intervention participants may not feel more compelled than the untreated control group to provide socially desirable response on weight, etc. Fifthly, the application of latent growth curve analyses allowed handling interindividual differences in trajectories of change in vegetable and fruit intake, body-mass index, and physical activity appropriately.

Several potential limitations should be discussed. Firstly, simple, widely used outcome measures were used instead of extensive self-report measures such as validated food-frequency or physical activity questionnaires, which may be more sensitive to change. For example, the assessment of physical activity did not distinguish between different intensity levels, and the assessment of diet with all its different components and macronutrients was limited to the assessment of the brief but widely used item on vegetable and fruit intake. These simple measures were a trade-in in favor of low patient selectivity, a core aspect of the public health impact of interventions [42] and a particular strength of this study. To ensure patients’ initial and ongoing participation over two years and as the interventions exclusively focused on alcohol use, assessments of secondary BRF outcomes had to be brief. For the same reason, objective measures such as hip or waist circumference or accelerometry were also not suitable. In addition, accelerometry can also bias measurement as it has been found to produce reactivity [46]. Secondly, data collection for this trial was finished 11 years ago, and technology has advanced since. However, while technology will continue to change, behavior change mechanisms addressed by this study’s highly individualized computer-generated feedback according to psychological behavior change theory remain to be core ingredients of effective behavior change interventions [32]. eHealth and mHealth interventions in healthcare require a solid theoretical foundation as tested in this trial informing developers about relevant psychological aspects of behavior change to be considered, and to achieve meaningful effects among patients. Core ingredients of our computer-generated intervention included proactivity and comprehensive stage-tailoring for high reach and stage-matched support. Thus, these rather explorative findings provide important insights into potential broader behavioral effects only obtainable from large RCTs, effective interventions, and long-term follow-ups as used in this study. Thirdly, findings may not be generalizable to patients excluded from the trial., e.g., 124 patients with indication of alcohol dependence were excluded as BAIs have been found to be insufficient [28], and the BAIs tested were specifically developed to address the majority of patients with non-dependent at-risk alcohol use. No severe limitations are expected with regard to the exclusion of patients too ill or without telephone due to small numbers (*n* = 11, *n* = 15), or to the low proportion of female participants. The latter is plausible given the lower prevalence of at-risk alcohol use among women than among men in the general population [6]. Trial participation and intervention efficacy were found to be unrelated to gender [17,47]. Fourthly, although overall rather high, follow-up engagement rates differed across study groups. A study on smoking cessation with similar methods, time-frames, and healthcare patients revealed that various methods of accounting for differential attrition bias did not change results [48]. To account for potentially different attrition mechanisms and to handle missing values adequately, our analyses were adjusted for all baseline variables that were at least marginally related to drop-out, and missing values were estimated using maximum likelihood estimation based on all available data. Fifthly, the study did not investigate whether the interventions worked differently well in patients with different medical conditions. However, our analyses were adjusted for medical wards, allowing conclusions for general hospital patients as a whole rather than for certain patient and disease subgroups only, which, from a preventive perspective, constitutes a further strength of the study.

## 5. Conclusions

Proactive, theory-driven BAIs seem to be able to produce beneficial effects concerning energy-balance-related BRFs among general hospital patients with at-risk alcohol use. These medium-sized additional beneficial and long-term BAI side effects with regard to body-mass index and potentially with regard to physical activity, beyond reduced alcohol use are particularly noteworthy given that they were achieved in people of whom the majority was little motivated to reduce their alcohol use to begin with. With respect to the prevention of chronic diseases, findings are encouraging concerning health behavior change interventions in healthcare given broader lifestyle change effects beyond the targeted behavior. Thus, our rather explorative findings underpin the need to further investigate additional beneficial BRF effects to better understand multiple BRF change following interventions. However, to achieve comprehensive effects concerning all BRFs, the development, evaluation, and implementation of proactive and individually tailored interventions targeting all BRFs seem to be needed in healthcare settings despite.

## Figures and Tables

**Table 1:** Observed participant characteristics concerning outcome variables at baseline and at all four follow-ups stratified by study group.

Outcomes		Baseline (N = 960)	Month 6 (N = 750)	Month 12 (N = 756)	Month 18 (N = 757)	Month 24 (N = 735)
Number servings vegetable and fruit per day (M, SD)						
Assessment only		2.0 (1.0)	2.0 (1.1)	-	2.1 (1.3)	2.1 (1.3)
In-person counseling		2.0 (1.2)	1.8 (1.1)		2.1 (1.2)	2.0 (1.1)
Computer-generated feedback		1.9 (1.2)	1.9 (1.2)		2.1 (1.1)	2.0 (1.2)
Number hours physical activity per week (*n*, %)						
Assessment only	None	100 (45.5)	113 (58.6)	-	115 (60.5)	110 (61.5)
	<1 h	40 (18.2)	9 (4.7)		11 (5.8)	10 (5.6)
	1–2 h	30 (13.6)	24 (12.4)		20 (10.5)	24 (13.4)
	>2 h *	50 (22.7)	47 (24.3)		44 (23.2)	35 (19.6)
In-person counseling	None	171 (48.3)	138 (52.1)		140 (51.1)	137 (51.1)
	<1 h	45 (12.7)	15 (5.7)		18 (6.6)	18 (6.7)
	1–2 h	51 (14.4)	30 (11.3)		37 (13.5)	31 (11.6)
	>2 h *	87 (24.6)	82 (30.9)		79 (28.9)	82 (30.6)
Computer-generated feedback	None	203 (52.5)	174 (59.6)		150 (50.7)	167 (57.6)
	<1 h	43 (11.1)	12 (4.1)		26 (8.8)	17 (5.9)
	1–2 h	51 (13.2)	38 (13.0)		42 (14.2)	36 (12.4)
	>2 h *	90 (23.2)	68 (23.2)		78 (26.3)	70 (24.1)
Body-mass index (M, SD)						
Assessment only		26.6 (5.4)	26.8 (5.2)	27.0 (5.2)	26.8 (5.1)	27.3 (5.3)
In-person counseling		27.0 (5.4)	27.6 (9.5)	27.0 (5.2)	27.1 (5.3)	26.9 (5.2)
Computer-generated feedback		27.0 (5.3)	27.3 (6.6)	26.9 (4.9)	27.2 (5.9)	27.1 (5.2)

Notes: - not measured; * to decrease complexity, categories 2–3 h, 3–4 h and >4 h were collapsed for these descriptive data.

**Table 2:** Mean differences in change in energy-balance-related BRFs from baseline to each follow-up between study groups.

	Baseline to Month 6		Baseline to Month 12		Baseline to Month 18		Baseline to Month 24		
	ΔM	95% CI	ΔM	95% CI	ΔM	95% CI	ΔM	95% CI	*p*
Vegetable and fruit intake per day									
In-person counseling vs. assessment only	0.001	−0.052; 0.055	0.003	−0.105; 0.110	0.004	−0.157; 0.166	0.006	−0.209; 0.221	0.958
Computer-generated feedback vs. assessment only	0.028	−0.023; 0.079	0.056	−0.046; 0.158	0.084	−0.068; 0.237	0.112	−0.091; 0.316	0.280
In-person counseling vs. computer-generated feedback	−0.027	−0.073; 0.020	−0.053	−0.146; 0.040	−0.080	−0.219; 0.059	−0.106	−0.292; 0.079	0.261
Physical activity per week									
In-person counseling vs. assessment only	0.069	−0.001; 0.139	-	-	0.208	−0.002; 0.418	0.277	−0.002; 0.557	0.052
Computer-generated feedback vs. assessment only	0.051	−0.015; 0.117	-	-	0.153	−0.045; 0.351	0.204	−0.060; 0.468	0.129
In-person counseling vs. computer-generated feedback	0.018	−0.046; 0.083	-	-	0.055	−0.139; 0.248	0.073	−0.185; 0.331	0.578
Body-mass index									
In-person counseling vs. assessment only	−0.119	−0.220; −0.017	−0.237	−0.440; −0.034	−0.356	−0.660; −0.052	−0.475	−0.880; −0.069	0.022
Computer-generated feedback vs. assessment only	−0.099	−0.198; 0.000	−0.198	−0.396; 0.000	−0.297	−0.595; 0.000	−0.396	−0.793; 0.000	0.050
In-person counseling vs. computer-generated feedback	−0.020	−0.104; 0.065	−0.039	−0.208; 0.130	−0.059	−0.312; 0.195	−0.078	−0.416; 0.259	0.650

Notes: Intercept and slope variances freely estimated; ΔM = mean difference in change; CI = confidence interval, *p* = *p*-value, - not measured.

## Data Availability

De-identified data analyzed for the study are not publicly available due to the German data protection laws but are available from the corresponding author on reasonable request that complies with the study purpose and the participants’ informed consent. The statistical code and other materials can also be accessed through the corresponding author.

## Supplementary Materials

The following supporting information can be downloaded at: https://www.mdpi.com/article/10.3390/healthcare14213590/s1, CONSORT 2025 Check list [15,49].
